# Hydrocortisone-induced parkin prevents dopaminergic cell death via CREB pathway in Parkinson’s disease model

**DOI:** 10.1038/s41598-017-00614-w

**Published:** 2017-04-03

**Authors:** Sangwoo Ham, Yun-Il Lee, Minkyung Jo, Hyojung Kim, Hojin Kang, Areum Jo, Gum Hwa Lee, Yun Jeong Mo, Sang Chul Park, Yun Song Lee, Joo-Ho Shin, Yunjong Lee

**Affiliations:** 1 Division of Pharmacology, Department of Molecular Cell Biology, Samsung Biomedical Research Institute, Sungkyunkwan University School of Medicine, Suwon, Gyeonggi-do 440-746 Republic of Korea; 2 Well Aging Research Center, DGIST, Daegu, 42988 Republic of Korea; 3 Companion Diagnostics and Medical Technology Research Group, DGIST, Daegu, 42988 Republic of Korea; 4 College of Pharmacy, Chosun University, Gwangju, 501-759 Republic of Korea; 5 Single Cell Network Research Center, Sungkyunkwan University School of Medicine, Suwon, Gyeonggi-do 440-746 Republic of Korea

## Abstract

Dysfunctional parkin due to mutations or post-translational modifications contributes to dopaminergic neurodegeneration in Parkinson’s disease (PD). Overexpression of parkin provides protection against cellular stresses and prevents dopamine cell loss in several PD animal models. Here we performed an unbiased high-throughput luciferase screening to identify chemicals that can increase parkin expression. Among promising parkin inducers, hydrocortisone possessed the most favorable profiles including parkin induction ability, cell protection ability, and physicochemical property of absorption, distribution, metabolism, and excretion (ADME) without inducing endoplasmic reticulum stress. We found that hydrocortisone-induced parkin expression was accountable for cell protection against oxidative stress. Hydrocortisone-activated parkin expression was mediated by CREB pathway since gRNA to CREB abolished hydrocortisone’s ability to induce parkin. Finally, hydrocortisone treatment in mice increased brain parkin levels and prevented 6-hydroxy dopamine induced dopamine cell loss when assessed at 4 days after the toxin’s injection. Our results showed that hydrocortisone could stimulate parkin expression via CREB pathway and the induced parkin expression was accountable for its neuroprotective effect. Since glucocorticoid is a physiological hormone, maintaining optimal levels of glucocorticoid might be a potential therapeutic or preventive strategy for Parkinson’s disease.

## Introduction

Progressive dopamine deficit in nigrostriatal circuit and loss of dopamine neurons are responsible for movement disorder in Parkinson’s disease (PD). Dysfunction of recessive PD gene parkin encoding E3 ubiquitin ligase has been implicated in PD^1–3^. As a multifunctional E3 ligase, parkin can mediate mono- and poly- ubiquitination (K48 or K63) of diverse substrates^4^. Lysine 48 polyubiquitination of parkin substrates has been widely investigated. Proteasomal targeting and degradation of parkin substrates following lysine 48 polyubiquitination regulate diverse cellular processes including poly(ADP-ribose) polymerase 1 (PARP1) dependent cell death^5^, mitochondrial biogenesis, antioxidant defense^6^, and mitochondrial quality control such as mitophagy and mitochondria motility^7, 8^.

PD-linked parkin mutations, nitrosylation, and phosphorylation by c-Abl can lead to its inactivation^2, 9–11^. Recently, it has been shown that parkin expression levels are decreased during the aging process, the most important risk factor in PD pathogenesis^12^. Conversely, overexpression of parkin in several PD animal models has been shown to be neuroprotective by restoring the nigrostriatal dopamine pathway^12–16^. Mechanisms of endogenous parkin expression and promoter regulation have been studied in an attempt to develop candidate neuroprotective agents. Mild endoplasmic reticulum stress can activate Activating Transcription Factor 4 (ATF4) that binds to parkin promoter, leading to parkin transcription^12, 17^. Several chemicals such as carbonyl cyanide m-chlorophenyl hydrazone (CCCP), thapsigargin, and the recently reported diaminodiphenyl sulfone can increase parkin levels via this process^17^. Although some of these chemicals have been suggested to be potential therapeutic lead compounds for PD via inducing parkin expression, elevated ER stress caused by them could be problematic since ER stress itself is one of causative factors in PD pathogenesis^18, 19^.

Here we attempted to identify parkin inducers based on high-throughput luciferase screening using parkin promoter luciferase reporter cell line. In this study, we found several candidate compounds such as hydrocortisone that could induce parkin expression and protect cells from oxidative stress with minimal or no ER stress. Hydrocortisone stimulated parkin expression in mouse brains and prevented dopamine neuron loss in 6-OHDA induced PD mouse models. We confirmed that parkin expression induced by hydrocortisone was responsible for cell survival against oxidative stress. We further showed that parkin expression induced by hydrocortisone was mediated by CREB signaling pathway. Pharmacological inhibition of glucocorticoid receptor abolished parkin expression induced by hydrocortisone, indicating that conventional cortisol signaling has a role in the maintenance of parkin expression. Overall, our results indicated that disruption of basal glucocorticoid pathway could be detrimental to the survival of dopamine neurons while hydrocortisone treatment could be potentially neuroprotective in dopamine neuron maintenance via inducing parkin in PD.

## Results

### High-throughput screening identifies parkin inducers

**Figure 1:**
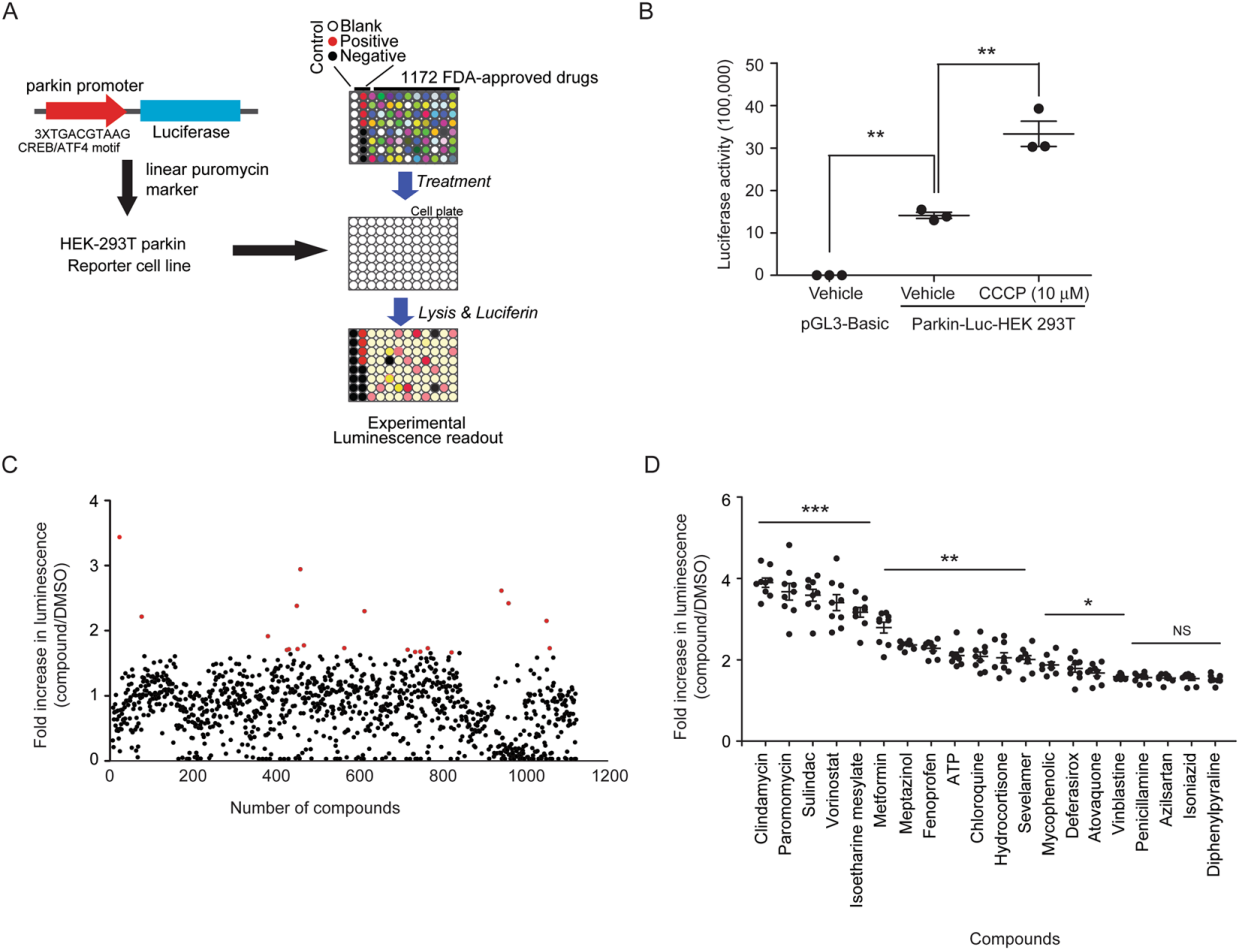
High-throughput luciferase screening to identify parkin inducing compounds. (**A**) Schematic illustration of the high-throughput screening method. HEK-293T parkin reporter cell line (parkin-Luc-HEK-293T) was selected by stable transfection of the luciferase construct containing three repeats of parkin promoter’s CREB/ATF4 binding motifs. In a 96-well plate, 1172 FDA-approved drugs were used to treat parkin-Luc-HEK-293T cells. Parkin promoter activity was measured by luciferase assay. DMSO was used as a negative control. CCCP treatment was used as a positive control. (**B**) Parkin promoter activities in parkin-Luc-HEK-293T cell line treated with DMSO or 10 uM CCCP as positive control (*n* = 4) were measured by luciferase assay. (**C**) Scatter plot showing relative increase of parkin promoter activity in parkin-Luc-HEK-293T cell line treated with each compound compared to DMSO negative control based on luciferase assay. Top 20 compounds were highlighted in red color. (**D**) Relative increase of parkin promoter activity induced by the top 20 compounds based on the initial HTS screening determined by luciferase assay (*n* = 9, see also Table 1). Quantified data are expressed as mean ± s.e.m. *P < 0.05, **P < 0.01, and ***P < 0.001, nonparametric Kruskal-Wallis ANOVA test (**B**) and ANOVA test followed by Tukey *post hoc* analysis (**D**).

To screen compounds that could increase parkin expression, we established a stable reporter HEK-293T cell line (Parkin-Luc-HEK-293T) by transfecting luciferase construct with parkin promoter elements containing three repeats of CREB/ATF4 binding motifs followed by selection with puromycin antibiotics. HEK-293T cells were chosen because they had low levels of basal parkin expression (Fig. 1A) suitable for parkin inducing compound screening. Based on luciferase assay, Parkin-Luc-HEK-293T reporter cell line exhibited basal parkin promoter activity compared to mock vector established cell line (Fig. 1B). Treatment with previously reported parkin inducer CCCP increased parkin promoter activity by more than two fold (Fig. 1B), validating the suitability of this reporter cell line for subsequent screening. In each 96-well plate, negative (DMSO treatment) and positive (CCCP treatment) controls were included to determine whether the assay was robust for high-throughput screening (HTS). Z-prime (Z’) factor value per plate was between 0.5 and 1, indicating that the response in this assay was large enough and the assay quality was good enough for HTS screening (Figure S1). For the identification of therapeutically applicable parkin inducers, 1172 FDA-approved drug chemical library (Selleck Chemicals) was used in the initial high-throughput luciferase screening in 96-well plate format. Parkin-Luc-HEK-293T cells were incubated with 10 µM of each compound for 48 hours. Parkin promoter activity was indirectly measured and plotted as fold increase of luminescence relative to that in DMSO treated sample (Fig. 1C). Top 20 compounds that increased parkin promoter activity in the initial screen were listed with its clinical implications (Table 1). Subsequent validation screening found that most of these compounds markedly elevated parkin promoter activities (Fig. 1D).

**Table 1:** List of parkin inducers identified by luciferase screening.

Compounds	Luc	Indication	Note
Vorinostat (SAHA)	3.4	Cancer	HDAC inhibitor
Deferasirox (Exjade)	2.9	Endocrinology	Iron chelator
Sevelamer HCl	2.6	Cardiovascular Disease	Binds to dietary phosphate
Chloroquine Phosphate	2.4	Inflammation	Anti-malarial agent
Hydrocortisone (Cortisol)	2.4	Infection	Steroid hormone
Mycophenolic (Mycophenolate)	2.3	Infection	Immunosuppressant
Vinblastine	2.2	Neurological Disease	Inhibits microtubule formation
Diphenylpyraline HCl	2.1	Neurological Disease	
Penicillamine (Cuprimine)	1.9	Immunology	Antirheumatic agent
Sulindac (Clinoril)	1.8	Cancer	Non-steroidal anti-inflammatory drug
Isoetharine Mesylate	1.7	Cardiovascular Disease	
Atovaquone (Atavaquone)	1.7	Neurological Disease	Used to treat or prevent for pneumocystis pneumonia
ATP (Adenosine-Triphosphate)	1.7	Endocrinology	
Metformin hydrochloride (Glucophage)	1.7	Endocrinology	Biguanide hypoglycemic agent
Paromomycin Sulfate	1.7	Infection	Aminoglycoside antibioticis
Clindamycin	1.7	Infection	Inhibits protein synthesis by acting on the 50S ribosomal.
Isoniazid (Tubizid)	1.7	Infection	Angiogenesis
Azilsartan (TAK-536)	1.7	Neurological Disease	Angiotensin II type 1 (AT1) receptor antagonist
Fenoprofen calcium hydrate	1.7	Immunology	Non-steroidal anti-inflammatory drug
Meptazinol HCl	1.6	Neurological Disease	Opioid analgesic

HEK-parkin-Luc reporter cells were treated with each compound. Parkin promoter activities were determined based on luciferase value (Luc.). They were normalized to that of DMSO control and expressed in the table (See also Fig. 1D). The clinical indication and note are adapted from the table provided by Selleck (L1300-Selleck-FDA-Approved-Drug-Library-1172cpd-Library).

**Figure 2:**
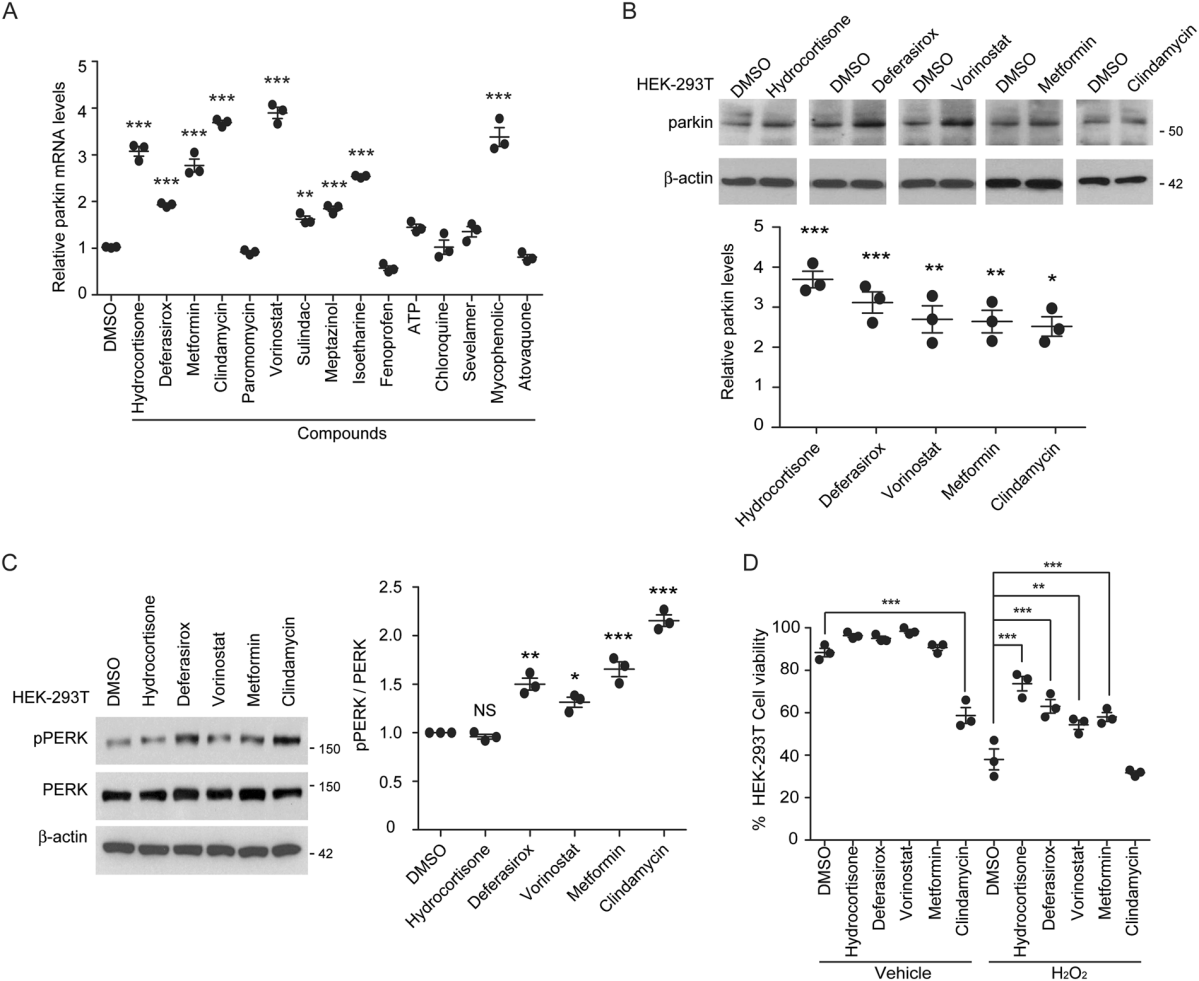
Parkin inducers prevent HEK-293T cell death induced by oxidative stress. (**A**) Real time quantitative PCR quantification of relative parkin mRNA levels in HEK-293T cells treated by the top 15 compounds selected from luciferase assay (Fig. 1D). Their levels were normalized to that of GAPDH as an internal loading control (*n* = 3). (**B**) Western blot analysis of parkin expression in HEK-293T cells treated with 10 µM of indicated compounds. DMSO was used as vehicle control (upper panel). Relative parkin protein levels in each compound-treated group were normalized to the level of β-actin. DMSO was used as a negative control for compound treatment (*n* = 3 independent experiments per group). (**C**) Representative western blot images of PERK and phosphorylated PERK (pPERK) in HEK-293T cells treated with the indicated parkin inducing compounds (left panel). Relative pPERK levels were normalized to total PERK levels (right panel) as an indicator of ER stress (*n* = 3 per group). (**D**) Viability of HEK-293T cells treated with indicated parkin inducing compounds and challenged with hydrogen peroxide (500 µM, 90 min) determined by trypan blue exclusion assay (*n* = 3 per group). Quantified data are expressed as mean ± s.e.m. *P < 0.05, **P < 0.01 and ***P < 0.001, unpaired two-tailed Student’s *t* test (**B**) or ANOVA test followed by Tukey *post hoc* analysis (**A**,**C**,**D**). Full blots (for cropped images in **B**,**C**) are presented in Figure S6.

Next, we determined whether the top 15 compounds that activated parkin promoter could indeed induce parkin expression. Several compounds (including hydrocortisone, deferasirox, metformin, clindamycin, vorinostat, sulindac, meptazinol, isoetharine, and mycophenolic acid) increased the expression levels of parkin mRNA in HEK-293T cells (Fig. 2A). In addition, the protein expression levels of parkin in HEK-293T cells were also increased after treatment with hydrocortisone, deferasirox, vorinostat, metformin, or clindamycin (Fig. 2B). Parkin expression level was increased 2- to 3- fold by these compounds (Fig. 2B), with the highest level increased by hydrocortisone. Notably, hydrocortisone did not increase PERK phosphorylation, an indicator of endoplasmic reticulum (ER) stress, whereas deferasirox, vorinostat, metformin, and clindamycin increased the ER stress indicator to some extent (Fig. 2C). Metformin and clindamycin increased ER stress levels more than 50% compared to DMSO control (Fig. 2C). It has been reported that parkin expression is critical to cell survival^12–16^. Therefore, we examined whether these parkin inducers could prevent oxidative stress-induced cell toxicity. Hydrogen peroxide treatment resulted in more than 50% reduction in cell viability of HEK-293T cells based on trypan blue exclusion assay (Fig. 2D), whereas treatment with hydrocortisone or deferasirox markedly increased the cell viability by 30% and 20%, respectively, against oxidative stress compared to DMSO control (Fig. 2D). Both metformin and clindamycin rendered mild resistance to HEK-293T cells against oxidative stress-induced cell death. However, clindamycin was toxic to HEK-293T cells. It killed HEK-293T cells by itself without hydrogen peroxide treatment (Fig. 2D). Taken together, hydrocortisone possessed the most favorable properties (parkin induction activity, no ER stress induction, and cell protection) for subsequent application in PD.

### Hydrocortisone induced parkin expression prevents cell toxicity induced by oxidative stress

**Figure 3:**
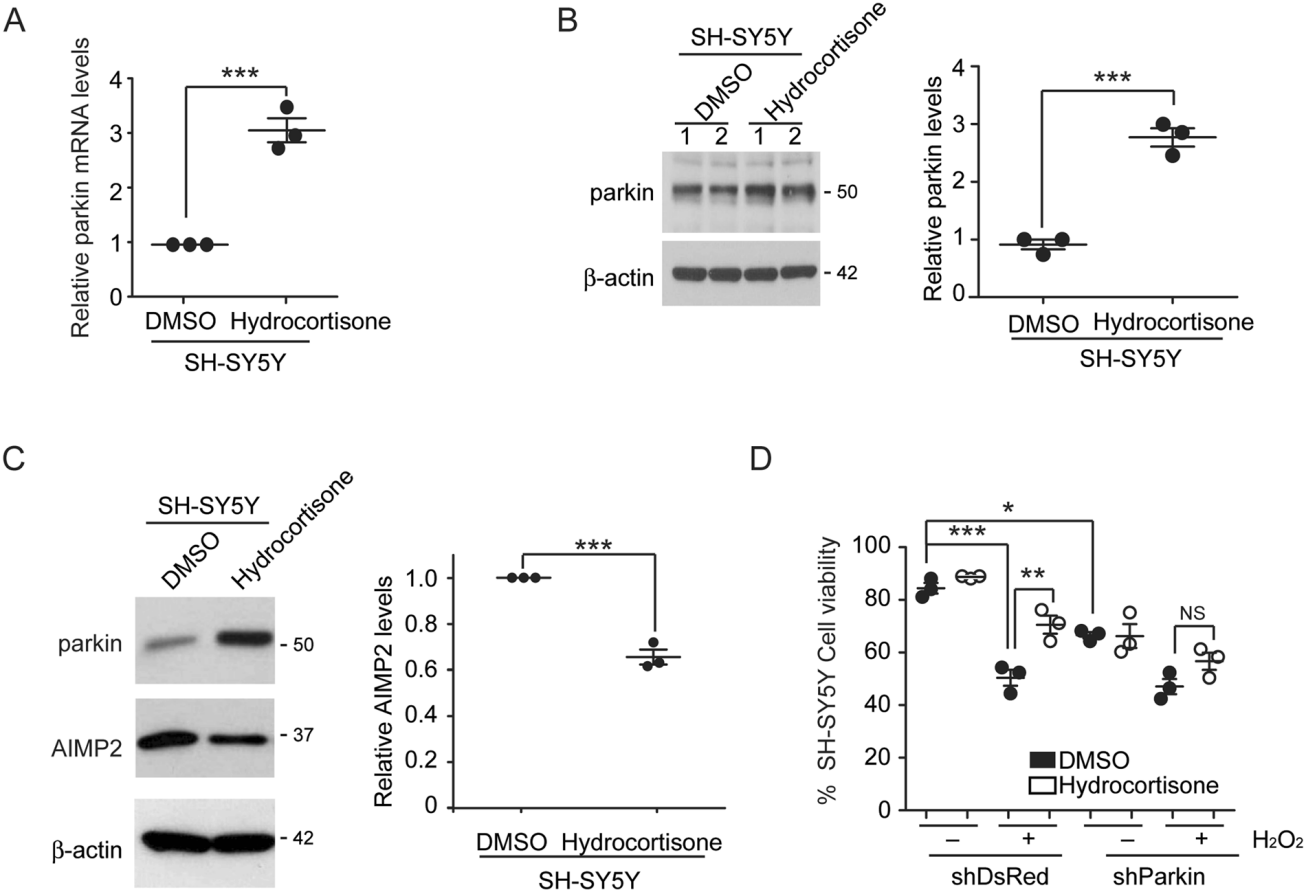
Hydrocortisone’s protection of SH-SY5Y cells is parkin dependent. (**A**) Parkin mRNA levels in SH-SY5Y cells induced by hydrocortisone (10 µM) treatment were normalized to the level of GAPDH based on real time quantitative PCR (*n* = 3). (**B**) Western blot analysis of parkin expression in SH-SY5Y cells treated with hydrocortisone or DMSO control (left panel). Relative parkin protein levels in SH-SY5Y cells treated with hydrocortisone or DMSO (right panel, *n* = 3) were normalized to that of β-actin. (**C**) Western blot analysis of parkin and its toxic substrate AIMP2 in SH-SY5Y cells treated with hydrocortisone or DMSO control (left panel). β-actin was used as an internal loading control. Relative AIMP2 protein levels (right panel, *n* = 3) were normalized to that of β-actin. (**D**) Viability of SH-SY5Y cells transiently transfected with shRNA to parkin or DsRed as control. Two days after transfection, SH-SY5Y cells were pretreated with hydrocortisone or DMSO and challenged with H_2_O_2_ (1 mM, 16 hrs). Cell viability was assessed by trypan blue exclusion assay (*n* = 3). Quantified data are expressed as mean ± s.e.m. **P < 0.01 and ***P < 0.001, unpaired two-tailed Student’s *t* test (**A**,**B**,**C**) or nonparametric Kruskal-Wallis ANOVA test (**D**). Full blots (for cropped images in **B**,**C**) are presented in Figure S6.

Following characterization of parkin inducers including hydrocortisone in HEK-293T cells, we next tested hydrocortisone in a neuroblastoma cell line SH-SY5Y. Similarly, parkin mRNA and protein levels in SH-SY5Y cells were elevated as much as three folds after treatment with hydrocortisone (Fig. 3A,B and C). Parkin is an E3 ubiquitin ligase that regulates its toxic substrates via lysine 48 linkage polyubiquitination. Among several parkin substrates, AIMP2 has been shown to be critical to PD because its accumulation can lead to dopaminergic cell death downstream of parkin inactivation^5, 11^. In this respect, we assessed whether parkin expression induced by hydrocortisone might alter the expression of AIMP2. Our results revealed that hydrocortisone-induced parkin elevation had correlation with marked reduction of AIMP2 levels in SH-SY5Y cells (40% reduction compared to that in DMSO control, Fig. 3C). Consistent with the observation of parkin induction and downregulation of toxic substrate AIMP2, treatment with hydrocortisone protected SH-SY5Y cells against cell death induced by hydrogen peroxide (Fig. 3D). Cell viability was improved 50% to 70% by hydrocortisone under oxidative stress condition. Cell protection by hydrocortisone was dependent on parkin induction because parkin knockdown by shRNA transfection (Figure S2) abolished the cell protection improved by hydrocortisone (Fig. 3D).

### Parkin expression induced by hydrocortisone is mediated by CREB

**Figure 4:**
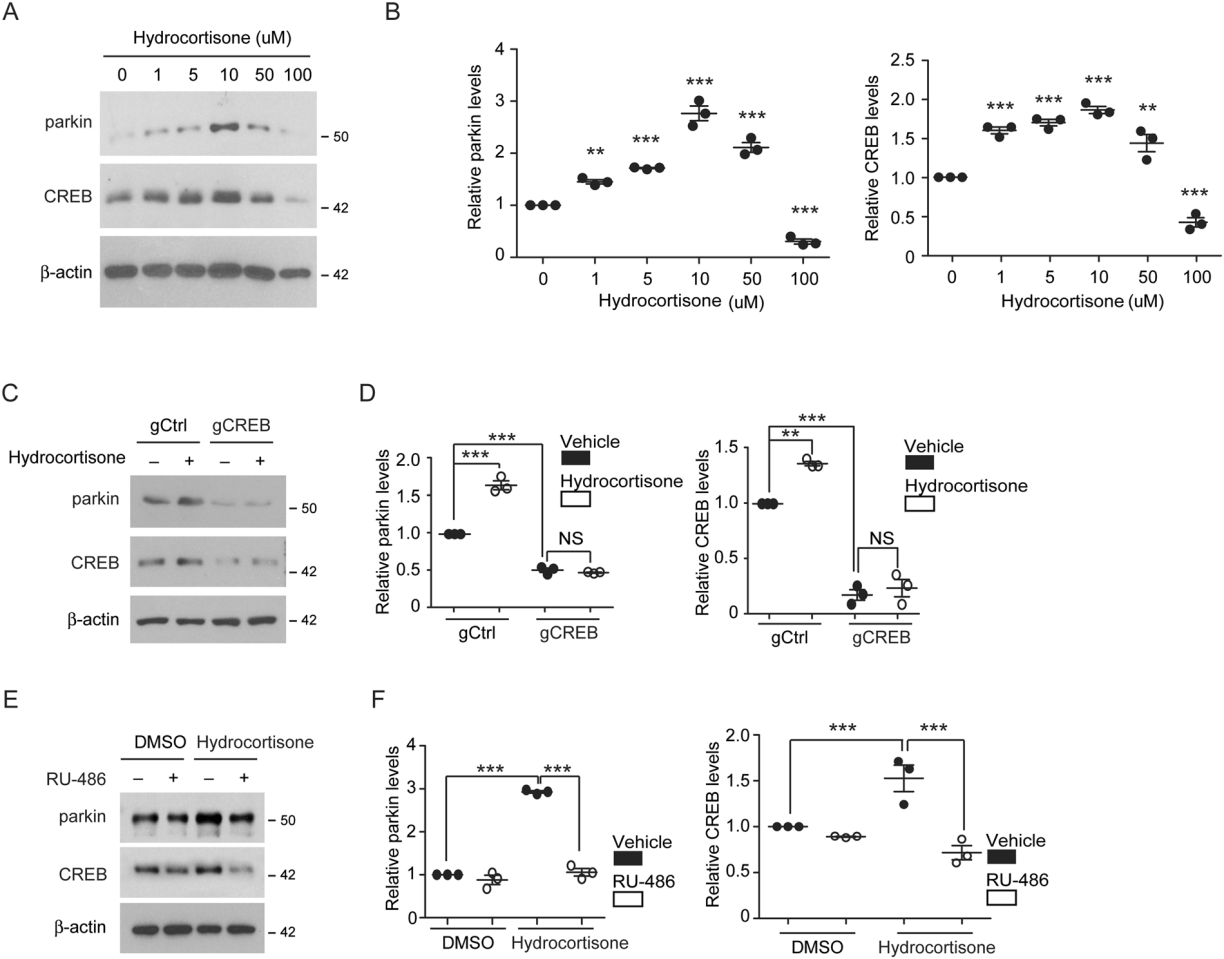
CREB-mediated parkin expression induced by hydrocortisone. (**A**) Expression of parkin and CREB in SH-SY5Y cells in response to treatment of hydrocortisone in increasing dose determined by western blot. (**B**) Relative levels of parkin or CREB were normalized to that of β-actin (*n* = 3). (**C**) Western blot analysis of parkin and CREB expression in SH-SY5Y cells transiently transfected with gRNA specific for CREB or control gRNA followed by hydrocortisone treatment. (**D**) Relative parkin or CREB levels were normalized to that of β-actin in the indicated experimental groups (*n* = 3). (**E**) Western blot analysis of parkin and CREB levels in SH-SY5Y cells. Cells were treated with glucocorticoid receptor antagonist RU-488 followed by hydrocortisone treatment to examine parkin and CREB induction. (**F**) Relative parkin and CREB induction in response to hydrocortisone treatment in SH-SY5Y cells pretreated with RU-486 (mifepristone) or vehicle control (*n* = 3 per group). Quantified data are expressed as mean ± s.e.m. **P < 0.01 and ***P < 0.001, ANOVA test followed by Tukey *post hoc* analysis (**B**,**F**, and parkin quantification in Fig. 4D) or nonparametric Kruskal-Wallis ANOVA test (CREB quantification in Fig. 4D). Full blots (for cropped images in **A**,**C**,**E**) are presented in Figure S6.

Next, we sought to determine the molecular mechanisms by which parkin was induced in response to hydrocortisone treatment. Parkin promoter contains ATF4/CREB binding motifs. Since several studies have confirmed that ER stress-induced ATF4 activation led to the transcription of parkin^12, 17^ and our results revealed that hydrocortisone did not increase PERK phosphorylation, we therefore hypothesized that CREB pathway might mediate parkin expression induced by hydrocortisone. With increasing concentrations of hydrocortisone treatment (1 µM to 100 µM), there were correlation between parkin expression levels and CREB levels (Fig. 4A,B). The levels of parkin and CREB were gradually increased by hydrocortisone treatment from 1 µM to 10 µM. Their levels reached the highest levels after treatment with 10 µM hydrocortisone. Hydrocortisone at 50 µM could still increase the expression levels of CREB and parkin compared to DMSO control. However, treatment with 100 µM hydrocortisone failed to increase the expression levels of parkin or CREB (Fig. 4A,B). The inability of high concentration of hydrocortisone to activate CREB/parkin pathway might be due to non-specific toxicity because 100 µM hydrocortisone treatment resulted in 40% reduction in cell viability of in SH-SY5Y cells (Figure S3). To further determine whether CREB expression was required for parkin expression induced by hydrocortisone, we knocked out endogenous CREB by transfecting SH-SY5Y cells with gRNA specific to CREB (CREB-gRNA). CREB-gRNA transfection resulted in efficient downregulation of endogenous CREB and blocked hydrocortisone-induced CREB elevation (Fig. 4C,D). Knockdown of CREB prevented parkin induction caused by hydrocortisone treatment (Fig. 4C,D), demonstrating that CREB expression mediated hydrocortisone-induced parkin expression in SH-SY5Y cells. Notably, ablation of endogenous CREB further reduced the basal expression level of parkin (Fig. 4C,D), indicating that basal CREB signaling pathway plays a role in the maintenance of physiological parkin expression. Hydrocortisone effect on CREB and parkin expression is mediated by specific binding to glucocorticoid receptor (GR) and activation of downstream signaling pathways because inhibition of GR with specific inhibitor (RU-486) in SH-SY5Y cells abolished CREB and parkin induction caused by hydrocortisone treatment (Fig. 4E,F). This notion is further supported by experiments using more potent GR agonist, dexamethasone. At the concentrations (1, and 10 nM) of no obvious cellular toxicity (Figure S4A), dexamethasone treatment increased the expression of parkin and CREB more than two folds in SH-SY5Y cells (Figure S4B–D). Together, these results indicate that basal activity of GR signaling and CREB pathway might be important for physiological parkin expression.

### Hydrocortisone prevents DA neurodegeneration in 6-OHDA PD model

**Figure 5:**
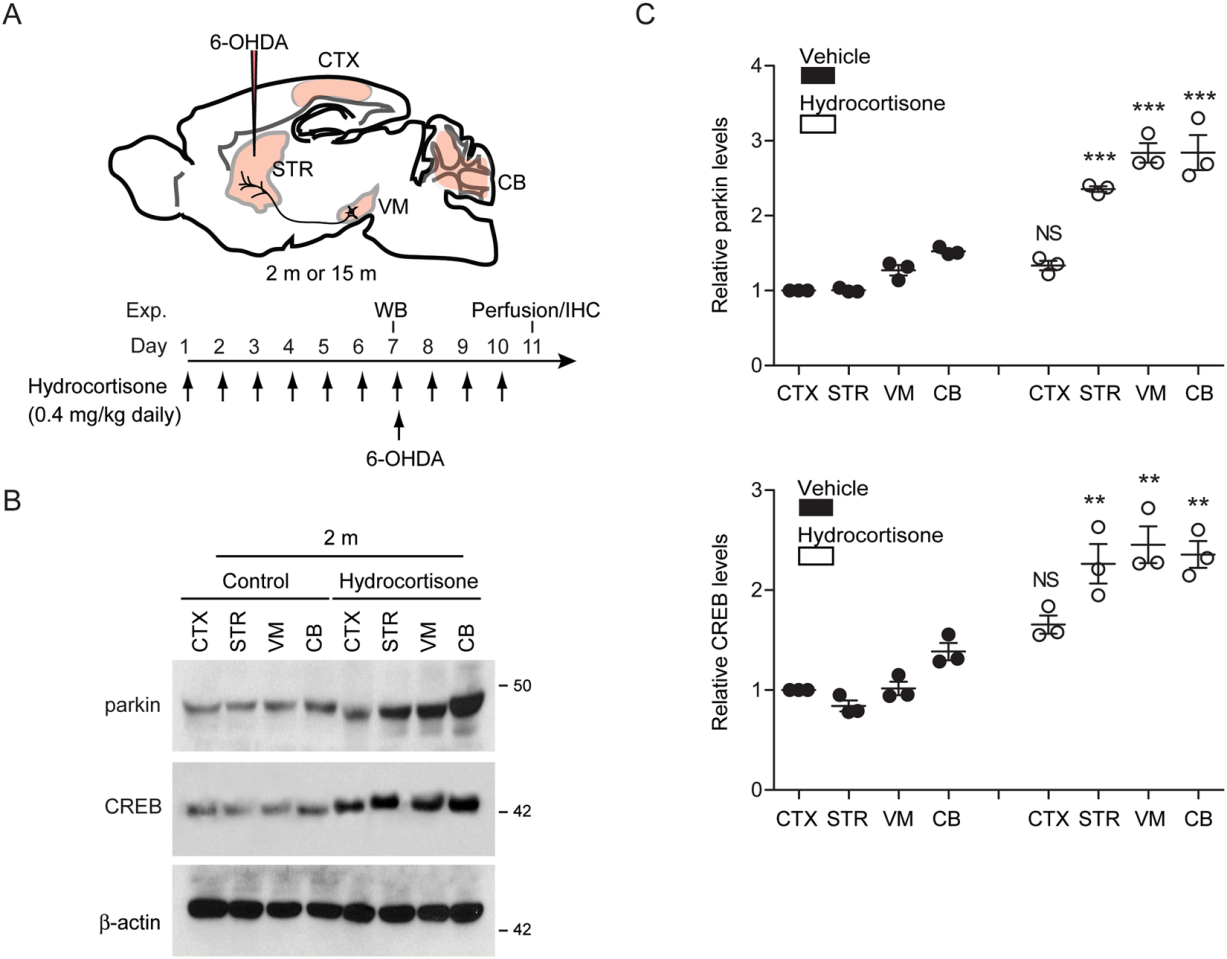
Hydrocortisone induces parkin *in vivo*. (**A**) A diagram illustrating the intrastriatal 6-OHDA injection and experimental schedule. WB: western blot; IHC: immunohistochemistry. Hydrocortisone (0.4 mg/kg body weight) was administered daily via intraperitoneal route. 6-OHDA was stereotaxically injected into the striatum of 2 months or 15 months old mice at day 7. Brain subregions dissected for western blot analysis were shaded in light pink (CTX: dorsal posterior parietal cortex; STR: striatum; VM: ventral midbrain; CB: cerebellum). (**B**) Parkin and CREB expression levels in the indicated brain subregions of mice treated with hydrocortisone or DMSO for 7 days were determined by western blot using parkin and CREB antibodies, respectively. (**C**) Relative parkin and CREB expression levels were normalized to that of β-actin in the brain subregions of mice (CTX: cortex; STR: striatum; VM: ventral midbrain; CB: cerebellum) treated with DMSO or hydrocortisone (*n* = 3 mice per group). Quantified data are expressed as mean ± s.e.m. *P < 0.05, **P < 0.01 and ***P < 0.001, unpaired two-tailed Student’s *t* test (**C**, parkin quantification) and nonparametric Mann-Whitney U test (**C**, CREB quantification). Full blots (for cropped images in **B**) are presented in Figure S6.

**Figure 6:**
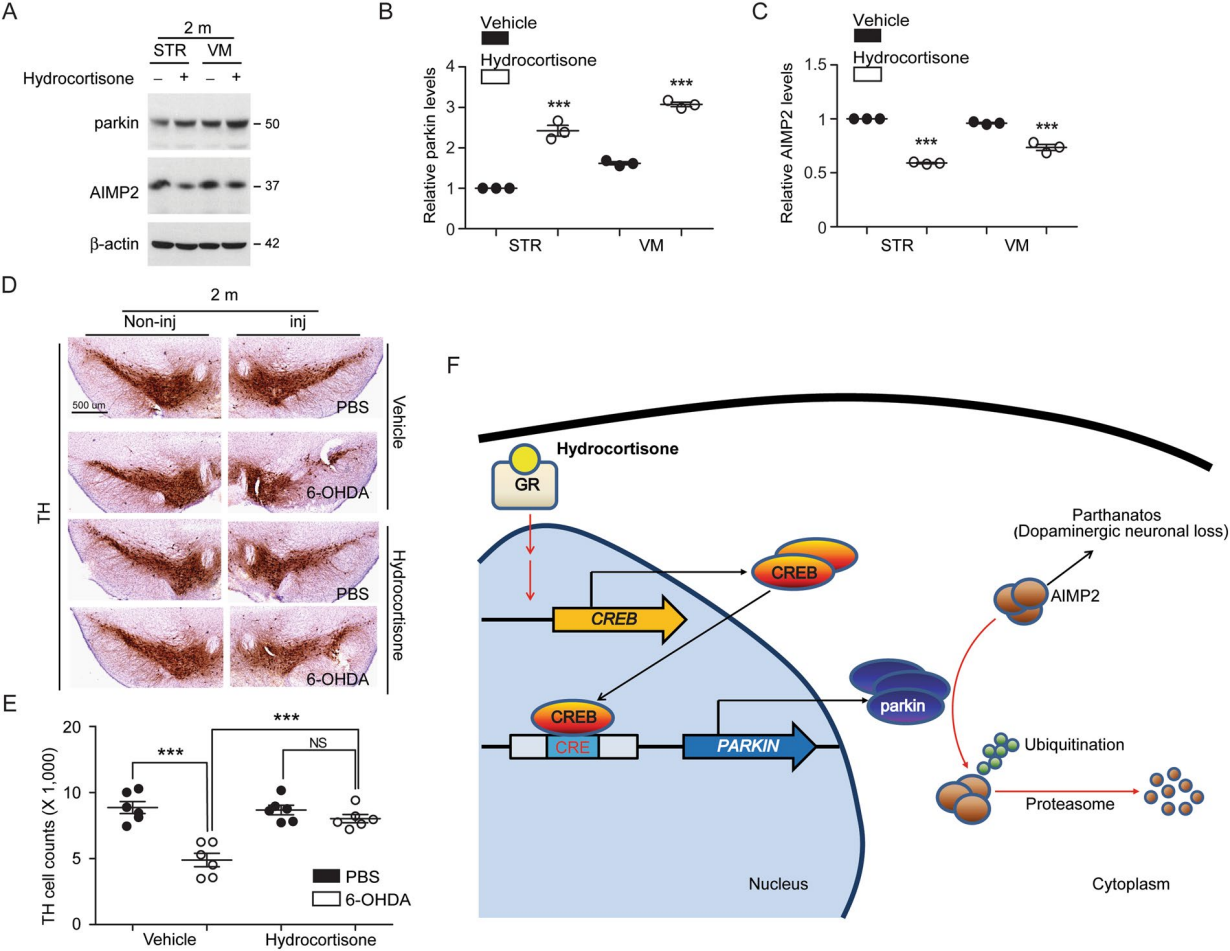
Hydrocortisone prevents dopamine cell loss in 6-OHDA mouse model. (**A**,**B**,**C**) Western blot analysis of parkin and its substrate AIMP2 in the striatum and ventral midbrain regions of mice (2 months old) treated with hydrocortisone or DMSO for 7 days. Relative parkin or AIMP2 protein levels were normalized to that of β-actin and shown as plot graphs (*n* = 3 mice per group). (**D**) Representative tyrosine hydroxylase (TH) immunohistochemistry (scale bar = 500 um) of the substantia nigra of 6-OHDA PD mouse models treated with hydrocortisone or DMSO. 6-OHDA (8 µg) was stereotaxically injected into the striatum (coordinate from bregma, L: −2.0, AP: 0.5, DV: −3.0 mm) to model dopaminergic neurodegeneration in mice. (**E**) Stereological assessment of tyrosine hydroxylase (TH)-positive dopaminergic neurons in the substantia nigra pars compacta of injection side from the indicated mouse groups (*n* = 6 mice per group). (**F**) Schematic summary of proposed molecular pathways illustrating hydrocortisone-mediated induction of parkin expression. Hydrocortisone binds to glucocorticoid receptor which in turn leads to expression of CREB. CREB increases parkin expression via binding to CREB binding motifs of parkin promoter region. Hydrocortisone-stimulated parkin expression results in the downregulation of the toxic parkin substrate AIMP2, which is beneficial for dopaminergic neuronal survival. Quantified data are expressed as mean ± s.e.m. ***P < 0.001, unpaired two-tailed Student’s *t* test (**B**,**C**) and ANOVA test followed by Tukey *post hoc* analysis (**E**). Full blots (for cropped images in **A**) are presented in Figure S6.

It has been shown that hydrocortisone can penetrate into the brain with diverse physiological actions through glucocorticoid receptor^20–23^. To determine whether hydrocortisone could increase parkin expression *in vivo*, we administered hydrocortisone intraperitoneally to mice for 7 consecutive days to examine parkin expression in mouse brains (Fig. 5A). Hydrocortisone administration resulted in approximate two-fold increase of parkin expression in selected brain subregions (striatum, ventral midbrain, and cerebellum) when compared to corresponding subregions of vehicle treated control mice (Fig. 5B,C). Consistent with the results in SH-SY5Y cells, there are correlating increases of CREB expression following hydrocortisone treatments *in vivo* (Fig. 5B,C). Hydrocortisone-stimulated parkin expression in the striatum and ventral midbrain (Fig. 6A,B, see also Fig. 5B,C) corresponded to the downregulation of parkin’s toxic substrate AIMP2 (20% downregulation, Fig. 6A,C).

Parkin expression has been shown to be protective against dopaminergic neuronal damage in several PD related animal models^6, 12–14, 16^. Therefore, we assessed the neuroprotective effects of hydrocortisone in 6-hydroxydopamine (6-OHDA)-induced PD mouse models. 6-OHDA injection into the striatum leads to retrograde and selective degeneration of nigrostriatal neurons over several weeks, and thus commonly used to model PD in mice^24^. Our results revealed that intrastriatal injection of 6-OHDA resulted in approximately 45% loss of tyrosine hydroxylase (TH)-positive dopaminergic neurons in the substantia nigra pars compacta (Fig. 6D,E). Hydrocortisone pretreatment in mice provided markedly enhanced dopaminergic neuronal survival against 6-OHDA intoxication. Dopaminergic neuronal viability was not affected by the administration of this low dose hydrocortisone *in vivo* (Fig. 6D,E).

To further determine translational impact of hydrocortisone, middle age female mice (15 months of age) were included for assessment of hydrocortisone’s protective effect and the same experimental procedure was applied (Fig. 5A). Similar to 2 month-old young male mice, hydrocortisone pretreatment to 15-month-old mice for 7 days led to an approximate two fold increase of parkin expression in the striatum and the ventral midbrain (Figure S5A,B). There was correlating reduction in the levels of the parkin substrate, AIMP2 in both brain subregions (Figure S5A,C). Stereological assessment in the substantia nigra pars compacta revealed that 6-OHDA intoxication resulted in about 65% loss of dopaminergic neurons which was largely prevented by hydrocortisone treatment (Figure S5D,E). Together these data indicate that both young and middle age mouse groups are responsive to hydrocortisone for beneficial effect on dopaminergic neuron survival and parkin expression.

## Discussion

As a neuroprotective E3 ligase, parkin has been shown to be critical for maintaining cellular functions for dopaminergic neuronal survival. Here, we identified several compounds that could increase parkin expression with potential function to prevent cellular toxicity following oxidative stress. The parkin promoter elements used in the establishment of luciferase reporter cell line have been initially characterized to be activated by ATF4 binding which is initiated by mild ER stress^12, 17^. Indeed, several compounds can activate these parkin promoter elements via ER stress induction. The extensive high-throughput luciferase screening in this study allowed us to obtain several parkin inducers, including hydrocortisone that increased parkin expression without inducing ER stress. Therefore, hydrocortisone might be used as promising therapeutic neuroprotective agent without causing ER stresses. It has been reported that hydrocortisone can suppress ER stress^25^ and the ATF4 signaling pathway in muscle tissues^26^. Hydrocortisone’s neuroprotective role by acting directly on neuronal cells has not been reported before, although there is a report showing that parkin induction in the frontal cortex by hydrocortisone might be implicated in Schizophrenia disorder^27^. Other parkin inducers including deferasirox, vorinostat, metformin, and clindamycin also elevated ER stress to some extent, consistent with previous reports on these compounds^28–30^. Iron chelator deferasirox and antidiabetic drug metformin have been proposed to be potential neuroprotective agents for the prevention of neurodegeneration. Since ER stress induced by them was modest but their parkin induction ability was substantial, deferasirox and metformin might merit further investigation to determine their potential as therapeutics for PD. Interestingly, antibiotic clindamycin strongly induced parkin expression. However, it failed to provide cell protection against oxidative stress probably due to the induction of ER stress based on PERK phosphorylation.

Our screening strategy provided a robust platform to identify novel parkin inducers. Z’ factor derived from negative and positive controls using the established Parkin-Luc-HEK-293T reporter cell demonstrated the robustness and readiness for high-throughput screening. Although we found potential parkin inducers that might serve as lead compounds to develop neuroprotectants in PD, larger chemical library could be employed for further high-throughput screening to identify more novel parkin inducers with better therapeutic properties.

Parkin induction via CREB pathway was discovered in this study for the first time (See Fig. 6F for graphic abstract of the proposed molecular pathway illustrating parkin induction by hydrocortisone). Hydrocortisone does not appear to bind directly to parkin promoter because parkin promoter is absent in the conserved binding motif of glucocorticoid receptor. Fortunately, the parkin promoter elements used in the luciferase assay possess motifs that could interact with ATF4 and CREB, which was why we initially hypothesized that CREB might be involved in parkin expression induced by hydrocortisone. Notably, it has been reported that dexamethasone treatment can enhance CREB expression potentially via indirect activation of CREB promoter through an unbiased genome wide screening^31, 32^. Consistent with their results, our data also support that both hydrocortisone and dexamethasone can increase CREB expression required for parkin promoter activation. In addition, knockout of basal CREB level by gRNA abolished endogenous expression of parkin, further supporting that CREB binding to parkin promoter is important to maintain basal parkin expression in the absence of stresses. Since GR signaling suppressed ATF4 signaling pathway, it would be instructive to study how CREB and ATF4 pathways would corroborate with each other to optimally regulate parkin expression depending on the environments that cells encounter.

It is interesting to note the region-specific effect of hydrocortisone for parkin induction *in vivo*. The part of cortex assessed in this study failed to show any induction of CREB and parkin expression which is in contrast to other brain subregions. This could be partly due to differential pharmacokinetic distribution of hydrocortisone, metabolism, and variable GR expression in the mouse brains^33^. Further investigation on cortisol concentration in the mouse brain subregions would be instructive in understanding regional difference of parkin/CREB induction by hydrocortisone.

Hydrocortisone exerts diverse effects on cellular function through GR activation. Upon binding of hydrocortisone, GR translocates to nucleus and binds to promoters of several genes depending on cells types^34, 35^. Therefore, it is expected that hydrocortisone treatment in cells would lead to alteration of diverse genes in addition to parkin. It is also possible that *in vivo* administration of hydrocortisone indeed can affect a lot of cell types including neurons and glial cells, thus preventing 6-OHDA induced dopaminergic neuronal death. Although genome wide gene expression analysis for each cell type would provide a complete understanding about hydrocortisone’s beneficial effects in Parkinson’s disease mouse model, it is obvious that parkin expression induced by hydrocortisone is required for cell protection against oxidative stress because knockdown of parkin background abolished hydrocortisone’s cell protection potential. It should be noted that parkin expression is maintained by GR/CREB signaling pathway. Pharmacological inhibition of GR blocked hydrocortisone-induced parkin expression. Interestingly, dopaminergic neurons of GR null mice are more vulnerable to mitochondrial toxin 1-methyl-4-phenyl-1,2,3,6-tetrahydropyridine (MPTP)^36^, suggesting that adequate physiological response to corticosteroid in the brain is critical for neuroprotection against toxic insults. Given that stress hormone cortisol is secreted daily on characteristic circadian clock or following impeding stresses, it is plausible that cortisol distribution to central nervous system (CNS) neurons might regulate parkin expression, thus controlling proper neuronal functioning and survival in response to environmental perturbation. One clinical report suggested the dysfunctional hypothalamic-pituitary-adrenal (HPA) axis in PD patients, resulting in lower plasma levels of adrenocorticotropic hormone (ACTH) and cortisol as compared to healthy age-matched controls^37^. Given the finding that cortisol mediates parkin expression, PD patients especially with dysfunctional HPA axis could be benefited from supplementation of hydrocortisone.

According to our results, hydrocortisone is considered as a potential preventive agent to increase parkin expression and confer resistance to cellular stresses in PD. Here, intrastriatal 6-OHDA injection mouse models were used to determine neuroprotective effect of hydrocortisone. Both male and female mice at the age of 2 months and 15 months respectively demonstrated similar responsiveness to hydrocortisone. Additional experiments using older mice and both genders would strengthen translational impact of this research since PD occurs on both genders at similar rates at old ages^38, 39^. Moreover, since 6-OHDA induced dopaminergic neurodegeneration and behavior deficit progresses over weeks^24, 40^, it would be informative to monitor hydrocortisone’s protective effect over prolonged period following 6-OHDA lesion.

One obstacle in the clinical application of hydrocortisone would be its known side effects (e.g., immunosuppression, high blood glucose and amino acid, hypertension, and psychosis), some of which are devastating^20, 41^. The dosage of hydrocortisone used to provide complete protection to dopaminergic neurons in our study was low compared to its dosage used to suppress immune response (0.4 mg/kg in our study vs. 20–100 mg/kg for anti-inflammatory effects in other studies^42–44^). Parkin induction and CREB activation were also achieved at lower concentration than the concentration of hydrocortisone that caused cellular toxicity. Based on our findings, it would be critical to have sufficient physiological supply of cortisol hormone to the CNS for protection of the brain. In this respect, hydrocortisone treatment could be considered as a supplementary measure to maintain parkin expression without causing serious side effects.

## Materials and Methods

### Chemicals and antibodies

FDA-approved drug library (cat# L1300, 100 µl/well, 10 mM solution in DMSO) was purchased from Selleck Chemicals LLC and used for high-throughput screening in this study. The glucocorticoid receptor antagonist, RU-488, and dexamethasone used in this study are from this library. Hydrocortisone and CCCP were purchased from Sigma. The following primary antibodies were used: mouse monoclonal antibody against parkin (cat# 4211, 1:5,000, Cell Signaling Technology), rabbit polyclonal antibody against pPERK (cat# 3179, 1:5,000, Cell Signaling Technology), rabbit polyclonal antibody against PERK (cat# 5683, 1:5,000, Cell Signaling Technology), rabbit polyclonal antibody against CREB (Cat# 9197, 1:5,000, Cell Signaling Technology), and rabbit polyclonal antibody against AIMP2 (cat# 10424-1-AP, 1:5,000, Proteintech). The following secondary antibodies were used: horseradish peroxidase (HRP)-conjugated sheep antibody against mouse IgG (cat# RPN4301, 1:5,000, GE Healthcare), HRP-conjugated donkey antibody against rabbit IgG (cat# RPN4101, 1:5,000, GE Healthcare), and HRP-conjugated mouse antibody against β-actin (cat# A3854, 1:10,000, Sigma-Aldrich).

### Cell culture and transfection

Human embryonic kidney cells (HEK-293T) and human neuroblastoma SH-SY5Y cells (ATCC, Manassas, VA) were grown in DMEM containing 10% (vol/vol) fetal bovine serum (FBS) and antibiotics. Cells were cultured at 37 °C in a humidified incubator supplied with 5% CO_2_/95% air. For transient transfection, cells were transfected with indicated constructs using X-tremeGENE HP transfection reagents (Roche) according to the manufacturer’s instructions. Unless otherwise indicated, lysates were prepared at 48 h post transfection.

### Plasmids

lentiCRISPR-gRNA to human CREB was constructed by ligating gRNA oligonucleotides into BsmBI restriction site of pLenti-CRISPR-v2 plasmid (Addgene). gRNA targeting sequence for hCREB was CACCGCTAATGTGGCAATCTGTGGC. pGL3-parkin-Luc construct^17^, pGL3 basic, pLKO-shRNA to parkin, and pLKO-shRNA to dsRed^6^ were described previously.

### Construction of parkin luciferase reporter cell line

HEK-293T cells in 6 well plates were transfected with pGL3-parkin-Luc construct (1 µg) or pGL3 basic (1 µg) and linear puromycin marker (0.2 µg, cat# 631626, Clontech). On the following day, transfected cells were passed onto ten 10 cm dishes with puromycin (5 ug/ml) containing media for selection. Puromycin resistant colonies were transferred onto 24 well pates using cloning discs (cat# Z374431, Scienceware cloning discs, 1/8 inches, Sigma-Aldrich) and further characterized to obtain parkin reporter cell line.

### Luciferase Assay

HEK-293T parkin reporter cells were harvested following treatments with each compound. Cell lysates were assayed for firefly luciferase activity using Dual Luciferase Reporter Assay System (Promega, Madison, WI) on a microplate luminometer (Berthold Technologies) according to the manufacturer’s instructions. Luciferase activity values after chemical treatment were normalized to the value of DMSO control.

### High-throughput luciferase screening

HEK-parkin-Luc reporter cells were plated into 96-well white flat-bottom plates at 80% confluency in 100 ul of DMEM containing 10% FBS with penicillin/streptomycin (P/S). On the next day, each compound (1172 FDA-approved drugs, Selleck Chemicals) was added to pre-warmed 50 ul DMEM at a final concentration of 20 uM. Half of the medium was replaced with pre-warmed DMEM containing the test compound. After 24 h incubation, luciferase activity was measured using SteadyGlo reagent (Promega). Each plate had multiple wells of CCCP positive controls (10 uM) and negative controls (0.1% DMSO). In order to assess HTS readiness and robustness of our assay, Z′ factor was quantified using the equation previously described^45, 46^. An HTS-ready assay should have a Z′ factor value of between 0.5 and 1^47^.

### Real-time quantitative PCR

Total RNA was extracted from cells using QIAzol Lysis Reagent (cat# 79306, QIAGEN) followed by DNase I treatment. cDNA was synthesized from total RNA (1.5 ug) using First-strand cDNA synthesis kit (iScript cDNA synthesis kit, Biorad). Ct values of each gene were obtained from SYBR green based realtime PCR reaction by using QuantStudio 6 flex Real-Time PCR System (Applied Biosystems). Relative mRNA expression levels of target genes were calculated by ΔΔCt method^48^ using GAPDH as an internal loading control. SYBR green PCR master mix (Cat# 4309155, Applied Biosystems) was used according to the manufacturer’s instructions. The following primers were used for real-time PCR amplification: GAPDH-F (AAACCCATCACCATCTTCCAG), GAPDH-R (AGGGGCCATCCACAGTCTTCT), parkin-F (CAGCAGTATGGTGCAGCGGA), parkin-R (TCAAATACGGCACTGCACTC).

### Trypan blue cell viability assay

SH-SY5Y cells were plated into 6-well plates at a seeding density of 0.5 × 10^6^ cells per well. Following transient transfection with indicated constructs, HEK-293T or SH-SY5Y cells were grown in DMEM containing low level of serum (2.5% FBS) with or without indicated chemicals for two more days. After hydrogen peroxide treatment or vehicle control, HEK-293T or SH-SY5Y cells were trypsinized, harvested and washed twice with PBS before resuspension in serum free DMEM. Resuspended cells were mixed with an equal volume of 0.4% trypan blue (wt/vol) and incubated at room temperature for 2 min. Live and dead cells were counted using automatic cell counter (EVE^TM^, NanoEnTek).

### Animal experiments

All animal experiments were approved by the Ethical Committee of Sungkyunkwan University in accordance with international guidelines. Male C57BL/6N mice (2 months old, total 30 mice were used in this study) were obtained from Orient (Suwon, Korea). Female C57BL/6N mice (15 months old, total 18 mice were used in this study) were kindly provided from Well Aging Research Center (DGIST). They were maintained with 12-h dark/light cycles in air-controlled rooms. Mice were provided *ad libitum* access to diet and water. All efforts were made to minimize animal suffering with minimum number of animals used. Hydrocortisone administration (0.4 mg/kg body weight, i.p.) began on day 1. Intrastriatal injection of 6-OHDA and stereological assessment were performed on day 7 and 11, respectively.

### Intrastriatal injection of 6-OHDA

For stereotaxic injection of 6-OHDA, eight-week-old or 15-month-old C57/BL6N mice treated either with hydrocortisone for 7 days or DMSO as a control were anesthetized with pentobarbital (60 mg/kg). We followed 6-OHDA injection procedure as described previously^24, 40^ with some modification. Briefly, an injection cannula (26.5 gauge) was applied stereotaxically into the striatum (anteroposterior, 0.5 mm from bregma; mediolateral, 2.0 mm; dorsoventral, 3.0 mm) unilaterally applied into the right hemisphere. Infusion was performed at a rate of 0.2 µl/min with 2 µl of 6-OHDA (4 µg/µl in sterile PBS) injected into each mouse. After the final injection, the injection cannula was maintained in the striatum for an additional 5 minutes for complete absorption of the chemical. It was then slowly removed from the mouse brain. Head skin was closed by suturing. Wound healing and recovery were monitored following the surgery.

### Preparation of tissues for immunoblot

Mice were euthanized by cervical dislocation. Mouse brain subregions (VM, STR, CB) were located following procedures described previously^49^. Dorsal posterior parietal cortex region (located above hippocampus; y coordinate from bregma = −1.7~−1.9) was dissected and used in this study and noted as CTX. Mouse brain tissues were homogenized in lysis buffer [10 mM Tris-HCl, pH 7.4, 150 mM NaCl, 5 mM EDTA, 0.5% Nonidet P-40, 10 mM Na-β-glycerophosphate, Phosphate Inhibitor Cocktail I and II (Sigma), and Complete Protease Inhibitor Mixture (Roche)] using a Diax 900 homogenizer. After homogenization, samples were rotated at 4 °C for 30 min for complete lysis. The homogenate was then centrifuged at 52,000 rpm for 20 min. Protein levels in the supernatants were quantified using BCA Protein Assay Kit (Pierce) with BSA as standards. Proteins were then subjected to immunoblot. Immunoblotting was performed with antibody of interest. Immuno-reactive bands were visualized with Enhanced Chemiluminescence Kit (Pierce). Densitometric analyses of protein bands were performed using ImageJ program (NIH, http://rsb.info.nih.gov/ij/).

### TH stereological cell counting

After scheduled treatments with hydrocortisone (0.4 mg/kg body weight, intraperitoneal administration daily) in 6-OHDA intoxication models or with DMSO control, animals were anesthetized with pentobarbital (50 mg/kg, intraperitoneal injection) and perfused with PBS followed by fixing with 4% paraformaldehyde (wt/vol in PBS). Brains were post-fixed with 4% paraformaldehyde overnight. They were subsequently cryoprotected in 30% sucrose in PBS (wt/vol) overnight. Every 4^th^ section (40 uM coronal sections) was used for analysis. Sections were incubated with anti-TH antibodies followed by sequential incubations with biotinylated goat anti-rabbit IgG and streptavidin-conjugated horseradish peroxidase (HRP) using Vectastain ABC kit (Vector Laboratories, Burlingame, CA) according to manufacturer’s instructions. 3,3-diaminobenzidine (DAB, cat# D4293, Sigma) was used as substrate of HRP to visualize TH positive cells. TH immunostained brain sections were counterstained with Nissl. Total numbers of TH-positive neurons in substantia nigra pars compacta were counted using Optical Fractionator probe of Stereo Investigator software (MicroBrightfield, Williston, VT). Experimenters were blinded for treatments of mice during stereological counting.

### Statistics

Power analysis was performed by using G*Power 3.1 software to determine approximate sample sizes for stereological counting. On the basis of mean difference from our preliminary experiments, a total sample size of four mice was calculated to obtain significant difference (effect size f = 22.42 for 45% mean difference; α = 0.05). Data are presented as mean ± standard error of mean (SEM). Normality of the data was tested with the Shapiro-Wilk test. Statistical significance was assessed either via unpaired two-tailed Student’s *t*-test and nonparametric Mann-Whitney *U* test for two-group comparison or analysis of variance (ANOVA) test with Tukey’s honest significant difference (HSD) post hoc analysis and nonparametric Kruskal-Wallis ANOVA test for comparison of more than three groups. *P* values less than 0.05 were considered statistically significant.

## Electronic supplementary material

Supporting Information
