# Supplementary material for: Hydrocortisone-induced parkin prevents dopaminergic cell death via CREB pathway in Parkinson’s disease model
Source: Sci Rep. 2017 Apr 3;7:525. doi: 10.1038/s41598-017-00614-w (PMC5428870; doi:10.1038/s41598-017-00614-w)
Supplement: Supplementary file 1 — Supporting Information [file 41598_2017_614_MOESM1_ESM.pdf]

Supporting Information

Hydrocortisone-induced parkin prevents dopaminergic cell death via CREB pathway in Parkinson's disease model

Sangwoo Ham, Yun-Il Lee, Minkyung Jo, Hyojung Kim, Hojin Kang, Areum Jo, Gum Hwa Lee, Yun Jeong Mo, Sang Chul Park, Yun Song Lee, Joo-Ho Shin, and Yunjong Lee

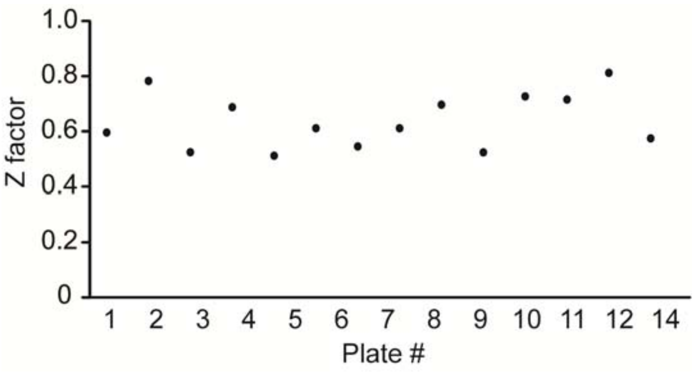

Figure S1. Z' factor of the high-throughput screening method of this study.

Scatter plot of Z' factor of each 96-well plate used in the high-throughput luciferase screening. Z' factors were calculated per each plate using values of means (M) and standard deviations (SD) from negative (DMSO) and positive control (10 μM, CCCP). $Z' = 1-3(SD_{CCCP}+SD_{DMSO})/(M_{CCCP}-M_{DMSO})$.

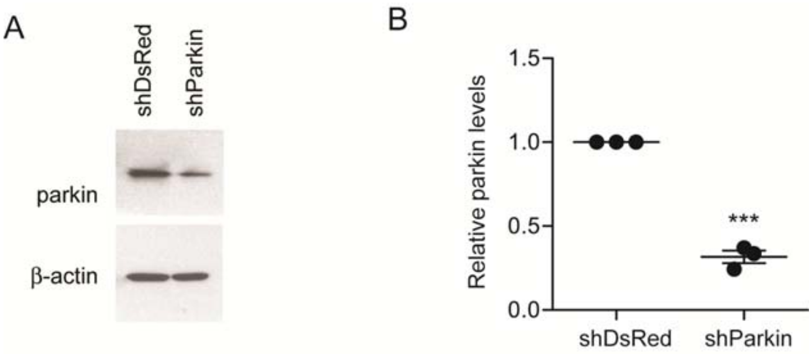

Figure S2. Efficient knockdown of parkin by shRNA.

(A) Efficient knockdown of parkin by transfecting shRNA specific to parkin in SH-SY5Y cells determined by western blot using indicated antibodies. β-actin was used as an internal loading control. (B) Relative parkin expression levels in SH-SY5Y cells transfected with shRNA to parkin were normalized to β-actin ($n = 3$). Quantified data are expressed as mean ± s.e.m. ***$P < 0.001$, unpaired two-tailed Student's *t* test.

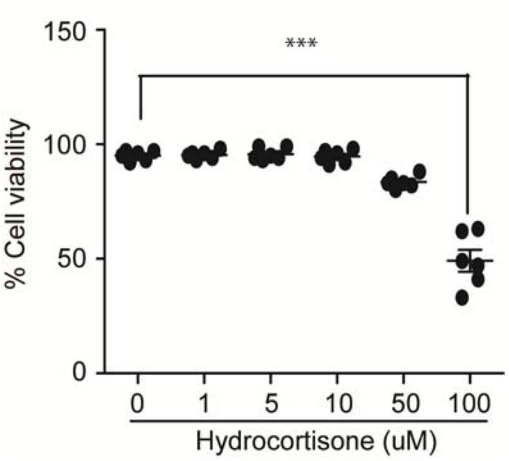

Figure S3. Toxicity of high dose hydrocortisone to SH-SY5Y cells.

Viability of SH-SY5Y cells treated with indicated dose of hydrocortisone was determined by trypan blue exclusion assay (n = 6 per group). Quantified data are expressed as mean ± s.e.m. ***$P < 0.001$, ANOVA test followed by Tukey *post hoc* analysis.

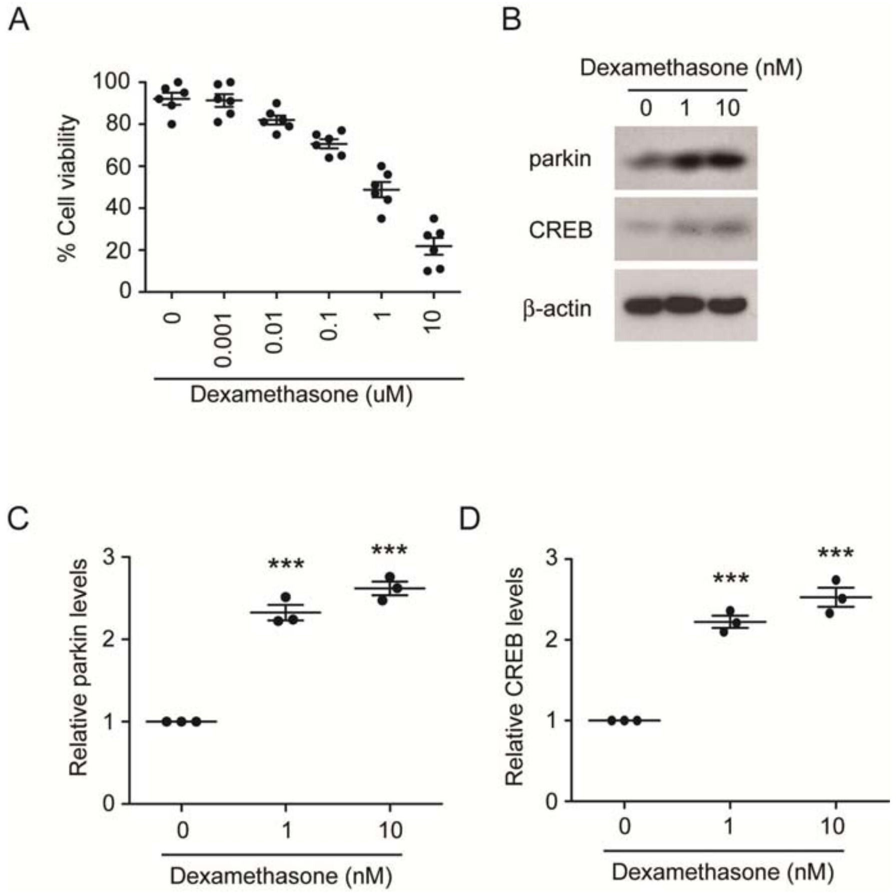

Figure S4. Dexamethasone increases CREB/parkin expression.

(A) Viability of SH-SY5Y cells treated with indicated dose of dexamethasone was determined by trypan blue exclusion assay (*n* = 6 per group). (B) Induction of parkin and CREB expression by dexamethasone treatment (0, 1, 10 nM, 24 hrs in SH-SY5Y cells determined by western blots. (C) Relative parkin expression levels in SH-SY5Y cells treated with dexamethasone (0, 1, 10 nM, 24 hrs) were normalized to β-actin (*n* = 3). (D) Relative CREB expression levels in SH-SY5Y cells treated with dexamethasone (0, 1, 10 nM, 24 hrs) were normalized to β-actin (*n* = 3). Quantified data are expressed as mean ± s.e.m. ***$P <$ 0.001, ANOVA test followed by Tukey *post hoc* analysis (C, D).

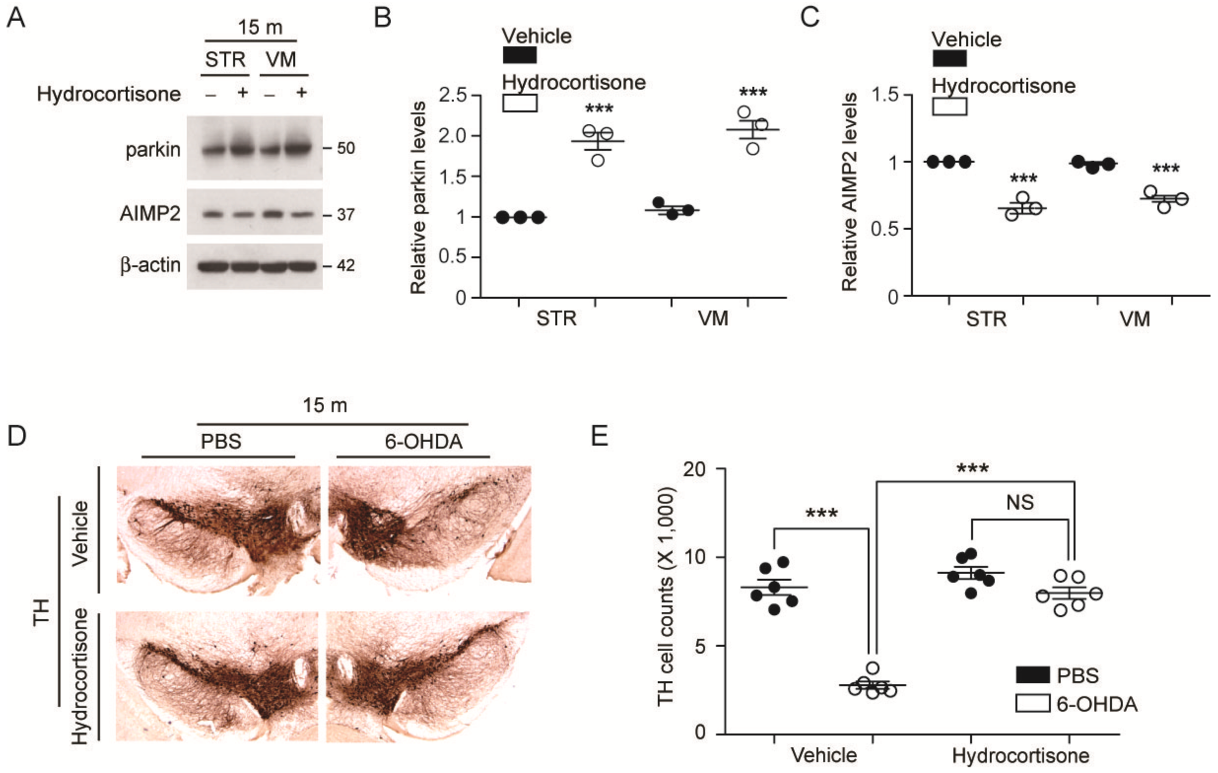

Figure S5. Hydrocortisone prevents dopamine cell loss in 15-month-old mouse models of 6-OHDA injection.

(A, B, C) Western blot analysis of parkin and its substrate AIMP2 in the striatum and ventral midbrain regions of mice (15 months old) treated with hydrocortisone or DMSO for 7 days. Relative parkin or AIMP2 protein levels were normalized to that of β-actin and shown as plot graphs (n = 3 mice per group). (D) Representative tyrosine hydroxylase (TH) immunohistochemistry of the substantia nigra of 6-OHDA PD mouse models (15 months old) treated with hydrocortisone or DMSO. 6-OHDA (8 µg) was stereotaxically injected into the striatum (coordinate from bregma, L: -2.0, AP: 0.5, DV: -3.0 mm) to model dopaminergic neurodegeneration in mice. (E) Stereological assessment of tyrosine hydroxylase (TH)-positive dopaminergic neurons in the substantia nigra pars compacta of injection sides from the indicated mouse groups (n = 6 injection sides per group. Total 12 mice were used for this study. PBS injeciton into the left striatum and 6-OHDA injection into the right striatum). Quantified data are expressed as mean ± s.e.m. ***P < 0.001, unpaired two-tailed Student's t

test (B, C) and ANOVA test followed by Tukey *post hoc* analysis (E).

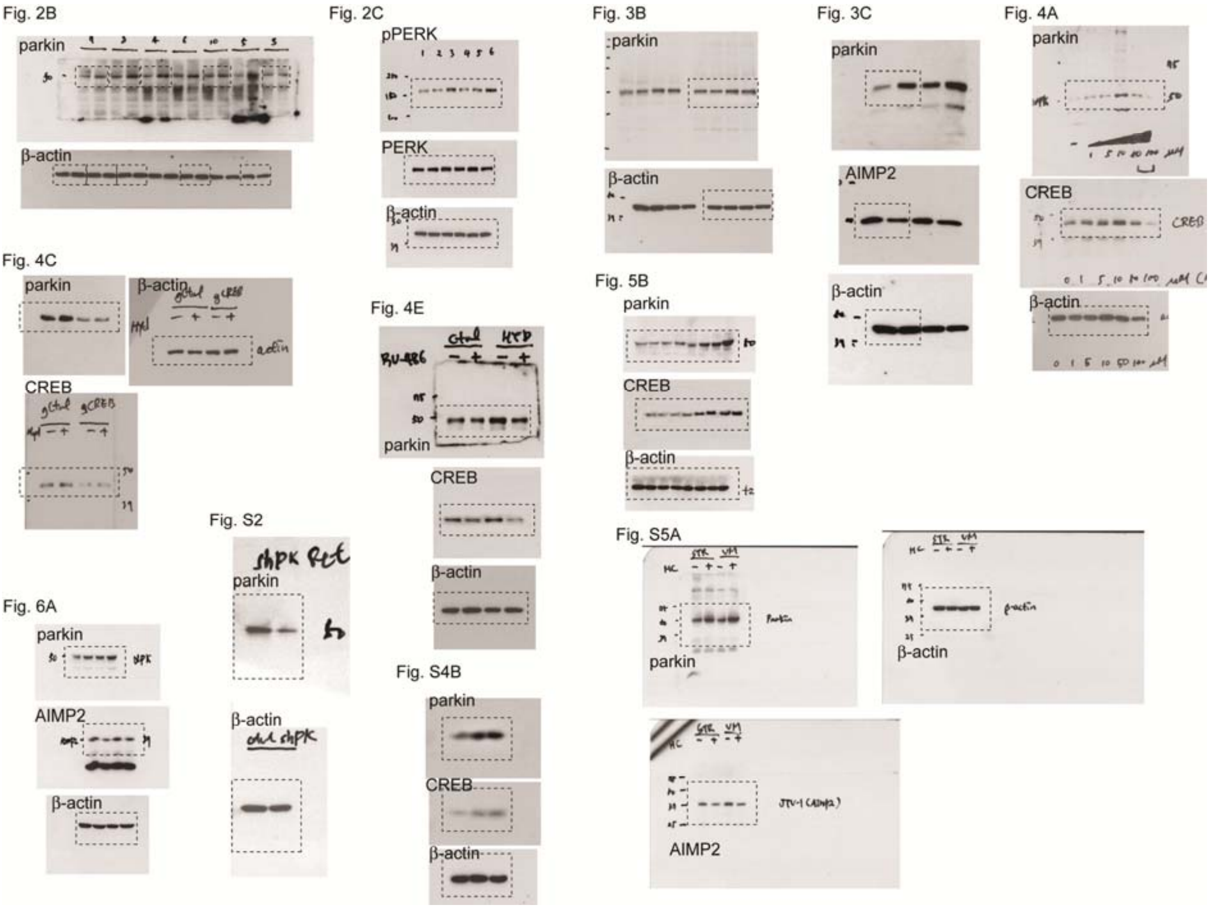

Figure S6. Full blot images of scanned western blots. Cropped regions used in figures are indicated with box with dotted lines. Each full blots are labeled to indicate the corresponding figure, panel and antibodies.
